# Supplementary material for: Epidemiology of paediatric gastrointestinal colonisation by extended spectrum cephalosporin-resistant Escherichia coli and Klebsiella pneumoniae isolates in north-west Cambodia
Source: BMC Microbiol. 2019 Mar 12;19:59. doi: 10.1186/s12866-019-1431-9 (PMC6417137; doi:10.1186/s12866-019-1431-9)
Supplement: Supplementary file 1 — Table S1. Sequence type distribution of E. coli isolates obtained in this study. Table S2. Sequence type distribution of K. pneumoniae isolates obtained in this study. (DOCX 17 kb) [file 12866_2019_1431_MOESM1_ESM.docx]

**Additional file 1**

**Table S1. Sequence type distribution of *E. coli* isolates obtained in this study**

| **Sequence Type** | **Frequency** | **Percent of total** |
| --- | --- | --- |
| ST38 | 10 | 10 |
| ST405 | 10 | 10 |
| ST131 | 9 | 9 |
| ST354 | 8 | 8 |
| ST648 | 7 | 7 |
| ST156 | 5 | 5 |
| ST410 | 4 | 4 |
| ST196 | 3 | 3 |
| ST44 | 2 | 2 |
| ST46 | 2 | 2 |
| ST48 | 2 | 2 |
| ST167 | 2 | 2 |
| ST394 | 2 | 2 |
| ST617 | 2 | 2 |
| ST2345 | 2 | 2 |
| ST5954 | 2 | 2 |
| ST101 | 1 | 1 |
| ST155 | 1 | 1 |
| ST226 | 1 | 1 |
| ST457 | 1 | 1 |
| ST469 | 1 | 1 |
| ST609 | 1 | 1 |
| ST1136 | 1 | 1 |
| ST1485 | 1 | 1 |
| ST1674 | 1 | 1 |
| ST1722 | 1 | 1 |
| ST2914 | 1 | 1 |
| ST3032 | 1 | 1 |
| ST3268 | 1 | 1 |
| ST4429 | 1 | 1 |
| ST4539 | 1 | 1 |
| ST6143 | 1 | 1 |
| ST6714 | 1 | 1 |
| **Novel ST** | **8** | **8** |
| 101-88-97-108-7-79-2 | 3 | 3 |
| 57-11-62-109-7-8-2  10-27-like (99.79%)-5-10-12-1-164 | 1  1 | 1  1 |
| 6-7-like (99.79%)-5-1-8-18-2 | 1 | 1 |
| 10-11-4-8-8-like (99.78%)-8-2 | 1 | 1 |
| 5-3-2-6-45-5-4 | 1 | 1 |
| **TOTAL** | **97** | **100** |

**Table S2. Sequence type distribution of *K. pneumoniae* isolates obtained in this study**

| **Sequence Type** | **Frequency** | **Percent of total** |
| --- | --- | --- |
| ST35 | 3 | 18 |
| ST17 | 1 | 6 |
| ST29 | 1 | 6 |
| ST22 | 1 | 6 |
| ST37 | 1 | 6 |
| ST48 | 1 | 6 |
| ST261 | 1 | 6 |
| ST273 | 1 | 6 |
| ST307 | 1 | 6 |
| ST334 | 1 | 6 |
| ST873 | 1 | 6 |
| **Novel ST** | **4** | **24** |
| 18-22-18-16-172-13-51  4-5-11-1-9-10-23 | 1  1 | 6  6 |
| 4-5-1-like (99.79%)-1-9-1-25 | 2 | 12 |
| **TOTAL** | **17** | **100** |
