# Supplementary material for: Epidemiology of paediatric gastrointestinal colonisation by extended spectrum cephalosporin-resistant Escherichia coli and Klebsiella pneumoniae isolates in north-west Cambodia
Source: BMC Microbiol. 2019 Mar 12;19:59. doi: 10.1186/s12866-019-1431-9 (PMC6417137; doi:10.1186/s12866-019-1431-9)
Supplement: Supplementary file 3 — Figure S1. Schematic of aligned genetic contexts for blaCTX-M-24 in study Escherichia coli. Features of interest are highlighted in the figure key. White numbers within open reading frames denote truncated sequence length (bp). Isolates harbouring this genetic context are listed to the left of the figure. “x” denotes contig breaks. P denotes plasmid contexts; c chromosomal contexts. (PDF 405 kb) [file 12866_2019_1431_MOESM3_ESM.pdf]

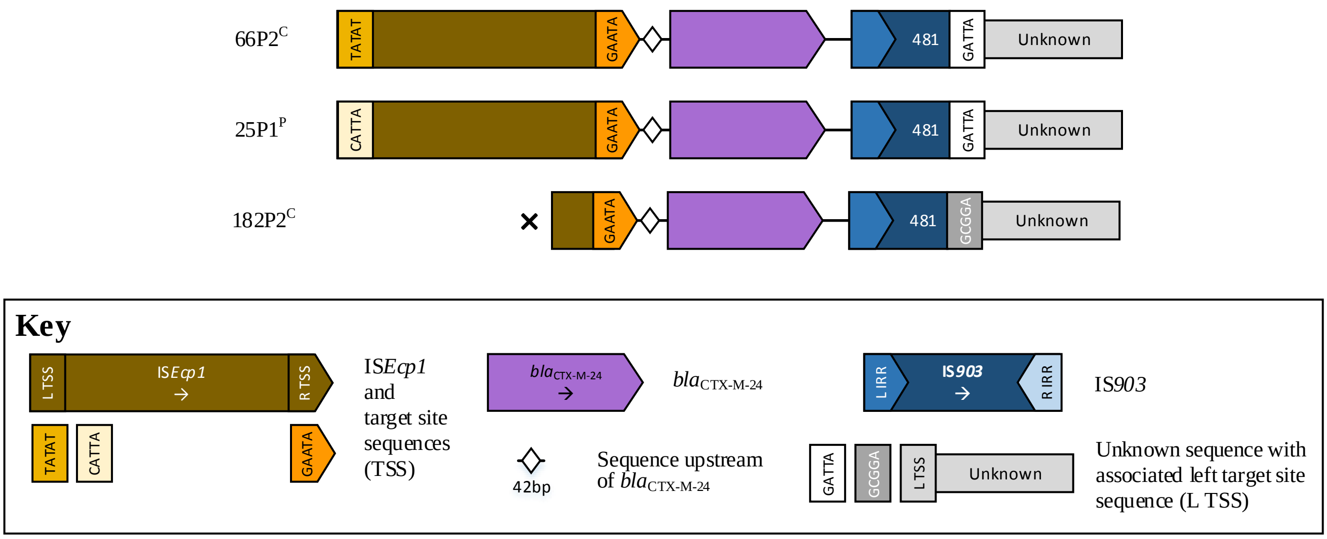

66P2C
TATAT
GAATA
481
GATTA
Unknown
25P1P
CATTA
GAATA
481
GATTA
Unknown
182P2C
GAATA
481
GCGGA
Unknown
Key
L TSS
ISEcp1
R TSS
ISEcp1 and target site sequences (TSS)
TATAT
CATTA
GAATA
blaCTX-M-24
blaCTX-M-24
42bp
Sequence upstream of blaCTX-M-24
L IRR
IS903
R IRR
IS903
GATTA
GCGGA
L TSS
Unknown
Unknown sequence with associated left target site sequence (L TSS)
