# Supplementary figures and images for: Epidemiology of paediatric gastrointestinal colonisation by extended spectrum cephalosporin-resistant Escherichia coli and Klebsiella pneumoniae isolates in north-west Cambodia
Source: BMC Microbiol. 2019 Mar 12;19:59. doi: 10.1186/s12866-019-1431-9 (PMC6417137; doi:10.1186/s12866-019-1431-9)

a) *E. coli*

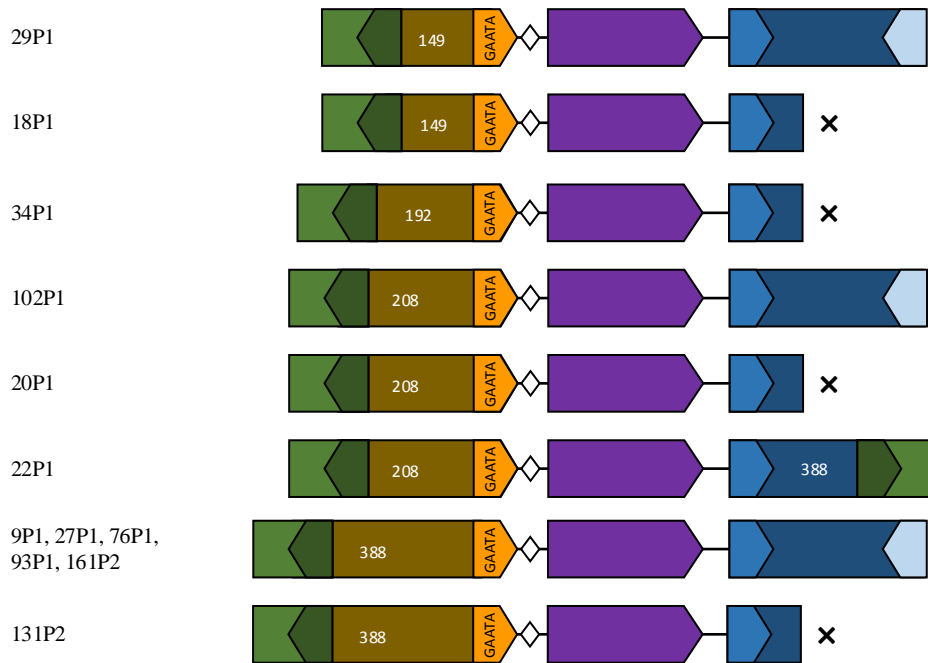

b) *K. pneumoniae*

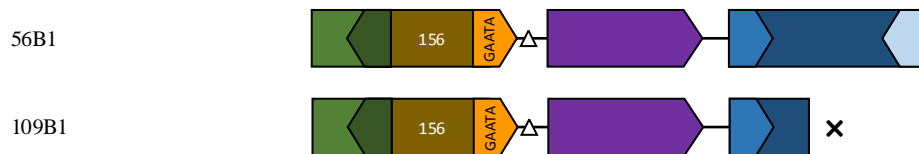

**Key**

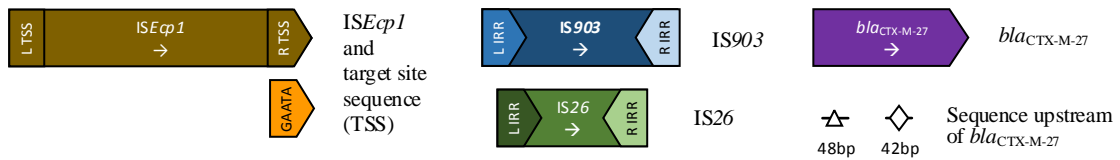

Supplement: Supplementary file 4 — Figure S2. Schematic of aligned genetic contexts for blaCTX-M-27 in study Escherichia coli and Klebsiella pneumoniae. Features of interest are highlighted in the figure key. White numbers within open reading frames denote truncated sequence length (bp). Isolates harbouring this genetic context are listed to the left of the figure. “x” denotes contig breaks. P denotes plasmid contexts; c chromosomal contexts. (PDF 410 kb) [file 12866_2019_1431_MOESM4_ESM.pdf]

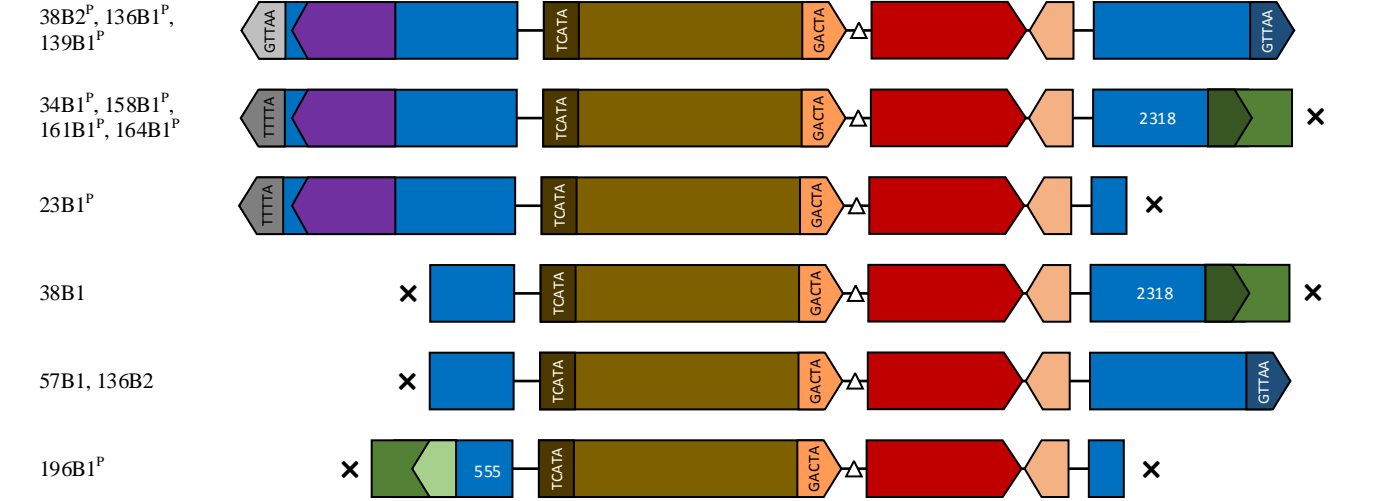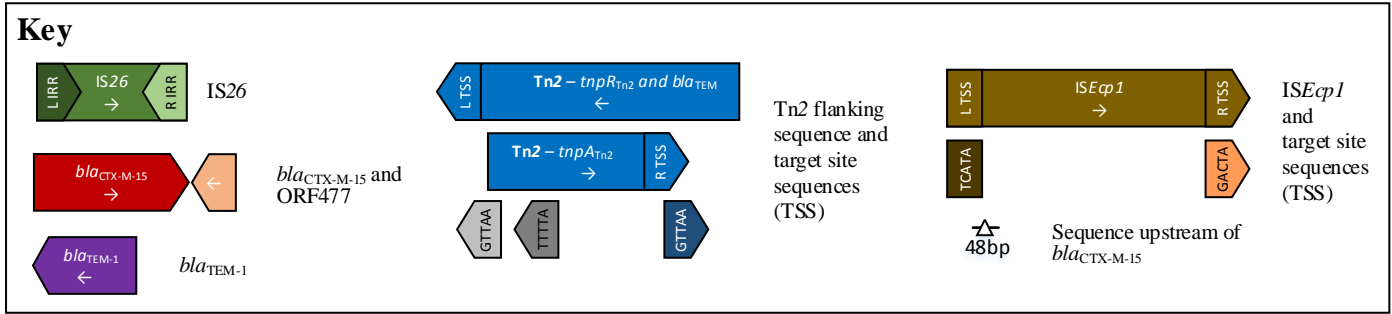

Supplement: Supplementary file 5 — Figure S3. Schematic of aligned genetic contexts for blaCTX-M-15 in study Klebsiella pneumoniae. Features of interest are highlighted in the figure key. White numbers within open reading frames denote truncated sequence length (bp). Isolates harbouring this genetic context are listed to the left of the figure. “x” denotes contig breaks. P denotes plasmid contexts; c chromosomal contexts. (PDF 410 kb) [file 12866_2019_1431_MOESM5_ESM.pdf]
